# Smoking induces sex-specific changes in the small airway proteome

**DOI:** 10.1186/s12931-021-01825-6

**Published:** 2021-08-24

**Authors:** Spela Kokelj, Jörgen Östling, Benjamin Georgi, Karin Fromell, Kristina Nilsson Ekdahl, Henric K. Olsson, Anna-Carin Olin

**Affiliations:** 1 Occupational and Environmental Medicine, School of Public Health and Community Medicine, Inst. of Medicine, Sahlgrenska Academy, University of Gothenburg, Box 414, 405 30 Gothenburg, Sweden; 2 PExA AB, Gothenburg, Sweden; 3 Translational Science and Experimental Medicine, Research and Early Development, Respiratory and Immunology, AstraZeneca, BioPharmaceuticals R&D, Gothenburg, Sweden; 4 Department of Immunology, Genetics and Pathology, Uppsala University, Uppsala, Sweden; 5 Linnaeus Centre for Biomaterials Chemistry, Linnaeus University, Kalmar, Sweden

**Keywords:** Small airways, Respiratory tract lining fluid, Exhaled particles, Proteomics, Smoking, Inflammation, COPD

## Abstract

**Introduction:**

Cigarette smoke triggers many cellular and signaling responses in the lung and the resulting inflammation plays a central role in smoke-related lung diseases, such as COPD. We explored the effects of smoking on the small airway proteome in samples obtained by collection of exhaled particles with the aim to identify specific proteins dysregulated by smoking.

**Methods:**

Exhaled particles were obtained from 38 current smokers, 47 former smokers and 22 healthy controls with the PExA method. 120 ng of sample was collected from individual subjects and analyzed with the SOMAscan proteomics platform. General linear model-based statistics were performed.

**Results:**

Two hundred and three proteins were detected in at least half of 107 total samples. Active smoking exerted a significant impact on the protein composition of respiratory tract lining fluid (RTLF), with 81 proteins altered in current smokers compared to never smokers (p < 0.05, q < 0.124). Among the proteins most clearly discriminating between current and never smokers were sRAGE, FSTL3, SPOCK2 and protein S, all of them being less abundant in current smokers. Analysis stratified for sex unveiled sex differences with more pronounced proteomic alterations due to active smoking in females than males. Proteins whose abundance was altered by active smoking in women were to a larger extent related to the complement system. The small airway protein profile of former smokers appeared to be more similar to that observed in never smokers.

**Conclusions:**

The study shows that smoking has a strong impact on protein expression in the small airways, and that smoking affects men and women differently, suggesting PExA sampling combined with high sensitivity protein analysis offers a promising platform for early detection of COPD and identification of novel COPD drug targets.

**Supplementary Information:**

The online version contains supplementary material available at 10.1186/s12931-021-01825-6.

## Introduction

More than 8 million deaths a year around the world are attributable to tobacco use [1]. Smoking is the most important risk factor for chronic obstructive pulmonary disease (COPD), with 15–36% of smokers developing COPD [2, 3]. The gaseous and particulate matter (tar) phase of cigarette smoke contain approximately 4700 different toxic substances, many of which are highly reactive oxygen/nitrogen species (RONS) [4]. Furthermore, a single puff of cigarette smoke is estimated to contain more than 10^14^ oxidizing molecules [5] and smoking one cigarette exposes the human respiratory tract to 10,000–40,000 μg of particulate matter (PM). These particles have a mean diameter of < 1 μm which allows a high rate of deposition in the distal parts of the human lung [6], especially in the transition zone of small airways. Free radicals, aldehydes, ketones and other species found in cigarette smoke may induce oxidative damage in many types of biological macromolecules, compromising their structure and/or function [7]. The inhalation of cigarette smoke triggers many cellular and signalling responses in the lung and the resulting inflammation plays a central role in smoke-related lung diseases [7]. The mechanisms by which smoking contributes to inflammation and tissue damage are, however, not completely understood [8]. Several proteomic studies applied to sputum, lung tissue and bronchoalveolar lavage (BAL) fluid from never, former and current smokers [7, 9] have identified smoke-induced alterations in proteins involved in the response to oxidative stress and inflammation as well as in extracellular matrix organization and wound healing [9, 10]. The complement system has been proposed an important driver of this inflammation [11] and its role in the pathogenesis of COPD is emerging [12, 13]. Several complement factors are synthesized locally in the lungs by the alveolar type II cells [14], providing a basis for local complement system activation and initiation of inflammation. The activation of the complement system leads to opsonisation, phagocytosis and recruitment of inflammatory cells to the infected or injured area [15]. Further, exposure to cigarette smoke has been reported to activate the alternative complement pathway [16], whereas inhibition of the alternative pathway is protective in preclinical models of lung injury [11].

In addition, analysis of lung epithelium gene expression has shown that whereas most smoking-related changes reverse following smoking cessation, some appear to be permanent, even in long-term former smokers [17–19].

It has also been reported that smoke-related pathogenic mechanisms in COPD may differ between women and men [20–23], with women appearing more susceptible to the adverse effects of smoking as compared to men [24]. Studies have shown that female smokers have a faster annual lung function decline [25] and a greater loss of FEV_1_ per pack-year of smoking [26], report a higher level of dyspnoea and are more likely to have bronchial hyperresponsiveness than men [27]. There is also a higher prevalence of females among patients with severe, early-onset COPD [28]. The increased susceptibility to cigarette smoke in women may have several aetiologies. First, there may be a genetic predisposition for smoking-related lung damage that is sex-specific [28]. Second possible explanation may be a dose-dependent effect. The airways of women are smaller and thus each cigarette represents a proportionately greater exposure [29]. Third, there may be hormonally mediated differences in tobacco-smoke metabolism, resulting in increased oxidative stress in female lungs [30].

The small airways may contribute substantially to the pathogenesis of COPD as they represent a “silent zone” where disease can progress over many years without causing symptoms and being detected [31]. The respiratory tract lining fluid (RTLF) is a protective interface between the respiratory epithelium and the external environment, and in small airways it consists mainly of lung surfactant, but also contains other biomolecules released from small airway epithelial cells [32, 33]. Changes in the small airway RTLF may reflect biological processes in this important lung compartment, which can be studied by collecting particles in exhaled air, a novel sampling method allowing non-invasive retrieval of biological material from the small airways [33–36]. The molecular composition of exhaled particles (PEx) has been explored previously and 124 different proteins were identified by LC/MS in pooled samples. A comparison of the identified PEx proteins with published bronchoalveolar lavage (BAL) proteomic data showed a high degree of overlap, with 103 (83%) of the PEx proteins having previously been detected in BAL [33].

In the present study we explored the effects of smoking on the small airway proteome in samples obtained by the collection of particles in exhaled air with the aim to identify dysregulation of specific proteins associated with smoking and to determine whether such changes are reversible following smoking cessation.

## Methods

### Study design and participants

One hundred and seven subjects with a median age of 61 years, (51% were women), were examined in 2016 and 2017. All subjects were recruited from our previous studies or by an advertisement in a daily paper. The cohort consisted of 38 current smokers (CS), 47 former smokers (FS) and 22 healthy never smokers (NS). The inclusion criterion for never smoking controls was post-bronchodilation FEV_1_/FVC > 0.70.

Subjects were defined as current smokers if they had smoked on a regular daily basis for at least one year at the time of examination. Former smokers were defined as those that had not smoked in the last 12 months but had smoked on a regular daily basis before that. Those who had never smoked on a regular basis were classified as never smokers.

Participants provided written informed consent prior to the measurements and the Regional Ethics Committee at the University of Gothenburg approved the study (442-17 and 390-06).

### Study assessments

All subjects filled out a questionnaire on medical history, symptoms and use of medication and a detailed smoking history was obtained, as well as information about second-hand exposure to cigarette smoke. Pack-years were calculated by multiplying the average number of packs smoked per day with the duration of smoking in years. Blood samples were obtained and analysed for hsCRP and white blood cell differential.

All subjects were instructed to withdraw from short-acting bronchodilators and long-acting bronchodilators at least 6 h and 24 h prior to the examination, respectively.

### Spirometry

Spirometry was performed using a Spirare spirometer (Spirare, Stockholm, Sweden) before and after bronchodilation with 400 µg of salbutamol in accordance with the ATS/ERS criteria [37]. Forced vital capacity (FVC), forced expired volume in one second (FEV_1_) an FEV_1_/FVC ratio were expressed as a percentage of the reference value (% pred) according to Brisman et al. (note the corrigendum) [38].

### Exhaled particles

Exhaled particles (PEx) were collected using the PExA instrument (PExA AB, Gothenburg, Sweden), as previously described [32, 39]. Study subjects breathed via a mouthpiece and a two-way, non-re-breathing valve into the instrument which consists of a thermostated box (36 °C) containing an optical particle counter (Grimm Aerosol Technik GmbH & Co, Ainring, Germany) and an impactor (Dekati Ltd, Tampere, Finland). The measured particle sizes cover diameters between 0.41 and 2.98 µm. Subjects inhaled HEPA-filtered air for a minimum of three breaths before the sampling in order to remove particles from ambient air. All subjects wore a nose clip throughout the procedure. A standardized breathing maneuver was used [35, 40], starting with an exhalation at normal flow rate to residual volume, breath holding for 5 s, followed by a maximal inhalation to total lung capacity, immediately followed by a normal exhalation to functional residual capacity. Exhalation flow was measured by an ultrasonic flow meter (OEM flow sensor; Spiroson-AS, Medical Technologies, Zürich, Switzerland), enabling visualization of the expiratory flow and volume [32]. Between breathing maneuvers, the subject breathed particle-free air tidally for 30 to 60 s. Each sampling session continued until 120 ng of exhaled particles were collected. After collection the sample holder was transferred to a clean air room and the substrate was cut out with a scalpel from the sample holder area and placed in Millipore Ultrafree-MC LH Centrifugal Filter insert (FC30LH25) and stored at − 80 °C for subsequent extraction and SOMAscan analysis. True blank samples were generated by applying the same sample handling procedure as for real samples but without collecting PExA sample from the study subjects.

### SOMAscan analysis and processing of data

PEx sample preparation is described in detail in Additional file 1. Prior to SOMAscan analysis, the volume of sample buffer was adjusted to reach the same concentration of PEx in all samples in order to normalize the samples for the differences in the collected amount of PEx.

SOMAscan (SomaLogic Inc, Boulder, USA) is an aptamer-based proteomics platform that uses slow off-rate modified DNA aptamers (SOMAmers) as high affinity protein capture reagents to simultaneously quantify more than 1300 human proteins in all types of protein extracts. The SOMAscan assay quantitatively transforms the proteins present in a biological sample into a specific SOMAmer-based DNA signal, expressed as relative fluorescent units (RFUs), which is directly proportional to the amount of target protein in the initial sample [41].

Intra-plate and inter-plate normalization were performed by SomaLogic according to their SOMAscan assay good laboratory practice (GLP) data quality-control procedures. Sample data was first normalized to remove hybridization variation within a run (Hybridization Control Normalization), followed by Calibration Normalization to remove assay differences between runs. SomaLogic’s data normalization procedures have been described previously in more details [42].

Limit of detection (LOD) was calculated as the mean plus 3 standard deviations based on two blank samples. Proteins with RFU values > LOD in more than 50% of the samples were considered for further analyses.

### Statistical analysis

Statistical analyses of the protein data were performed using general linear model-based statistics (Qlucore Omics Explorer 3.6 software, Qlucore AB, Lund, Sweden). SOMAscan data was log_2_ transformed before the analysis to achieve normal distribution. General linear model (GLM), with each variable normalized to mean 0 and variance of 1, was used to determine differences in protein abundance between NS, FS and CS and all the analyses were adjusted for the investigator performing the PExA measurements, as well as the age of the subjects. Statistical analyses are described in more details in Additional file 1. Group comparisons of SOMAscan data were considered hypothesis free and proteins with p-value < 0.05 were considered to be of interest in this explorative study. Linear regression was used to assess correlations between pack-years, time since smoking cessation and protein levels. Statistical analysis of clinical and demographic data was performed using IBM SPSS Statistics for Windows, version 26 (IBM Corp., Armonk, N.Y., USA) with the significance level set to p < 0.05.

## Results

### Demographic and clinical characteristics

Female former smokers had significantly lower post-FEV_1_/FVC and post-MMEF/FVC ratio compared to never smokers, while no significant difference was observed between female current and never smokers. The results also indicated that post-FEV_1_/FVC and post-MMEF/FVC ratio was lower in male current and former smokers compared to never smokers. The number of pack-years in former smokers was significantly lower compared to current smokers both in males and females. A higher number of blood neutrophils was observed in CS compared to NS and FS in males. Similar pattern was observed in females, although the difference did not reach statistical significance (Table 1).

**Table 1:** General characteristics and clinical data of the subjects included in the study

	Never smokers		Former smokers		Current smokers		p-value	
	Female	Male	Female	Male	Female	Male	Female	Male
Group size (N)	13	9	25	22	17	21	NA	NA
Age (years)	51	56	60	63	62	62	**0.020**	0.074
BMI (kg/m^2^)	24.5	25.0	25.9	26.3	26.3	26.0	0.505	0.225
Pack-years	0	0	23	22	38	39	**0.044**^**c**^	**0.004**^**c**^
Years since cessation of smoking	NA	NA	11	19*	NA	NA	NA	NA
GOLD (I/II)	0	0	7/2	5/3	2/0	1/6	NA	NA
FVC % pred^a^	96.7	104.2	97.0	93.9	91.3	96.6	0.383	0.221
FEV1% pred^a^	95.1	96.6	91.7	87.2	91.5	87.0	0.064	**0.032**
FEV1/FVC^a^ (%)	79.3	77.7	73.9	73.3	77.9	72.4*	**0.022**	0.055
MMEF/FVC^a^ (%)	76.0	71.9	47.1	51.9	70.4	52.1*	**0.009**	0.063
hsCRP (mg/L)	0.7	1.0	0.9	1.0	1.4	1.4	0.155	0.754
Neutrophils (1.8–7.5 × 10*9/L)	3.7	3.3	3.3	3.2	4.2	4.8	0.098	**0.005**
Lymphocytes (0.8–4.5 × 10*9/L)	1.9	1.5	1.8	1.6	2.2	2.2	0.074	**0.011**
Monocytes (0.1–1.0 × 10*9/L)	0.40	0.30	0.40	0.40	0.40	0.50*	0.707	**0.019**
Eosinophils (0.04–0.4 × 10*9/L)	0.10	0.10	0.10	0.10	0.20	0.20	0.496	0.136
Number of PEx per breath (kN/breath)	86.8	111.6	58.1	56.2	90.7	63	**0.035**	0.192

Median values are presented. p-values are based on a non-parametric Kruskal–Wallis test comparing smoking categories and stratifying for sex

*NA* not applicable, *PEx* exhaled particles, *kN/breath* thousand number of exhaled particles per breath

^a^Post-bronchodilation

^c^p-value based on Mann–Whitney test between former and current smokers

*Statistically significant difference (p < 0.05) between females and males based on Mann–Whitney test

### Effects of smoking on small airway proteome

**Fig. 1:**
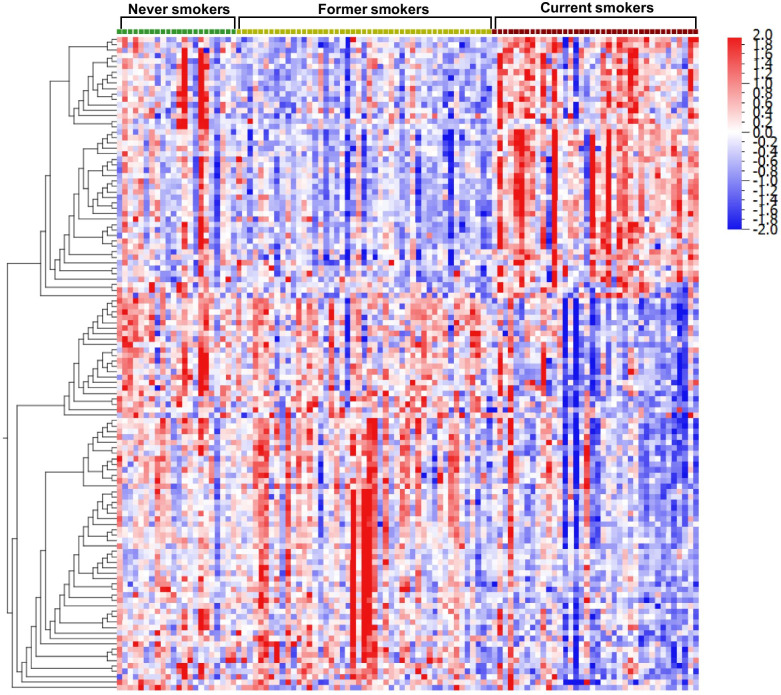
Clustering analysis of proteins based on smoking status. The 120 proteins whose abundance levels differ between never, former and current smokers (p < 0.05) were clustered by hierarchical clustering and the samples were ordered by smoking status. Protein abundance levels were adjusted for the effects of age and investigator

All in all, 203 proteins were detected in at least 50% of the 107 samples (Table S1, Additional file 2). A comparison of proteins detected in our samples with previously published SOMAscan BAL proteomic data showed a high degree of overlap, with 180 (89%) proteins having previously been detected in BAL with the SOMAscan analysis [43]. Using GLM-based statistics and adjusting for age and variance introduced by the investigator revealed statistically significant differences in 120 proteins between at least two of the groups (q < 0.05). As demonstrated by clustering analysis based on smoking status, these proteins mainly distinguished CS from FS and NS (Fig. 1).

Linear regression (adjusted for age) in FS and CS revealed a significant correlation between the relative abundance of 70 proteins and number of pack-years (p < 0.05, ∣R∣ ≥ 0.22).

### Current smokers versus never smokers and sex differences

**Fig. 2:**
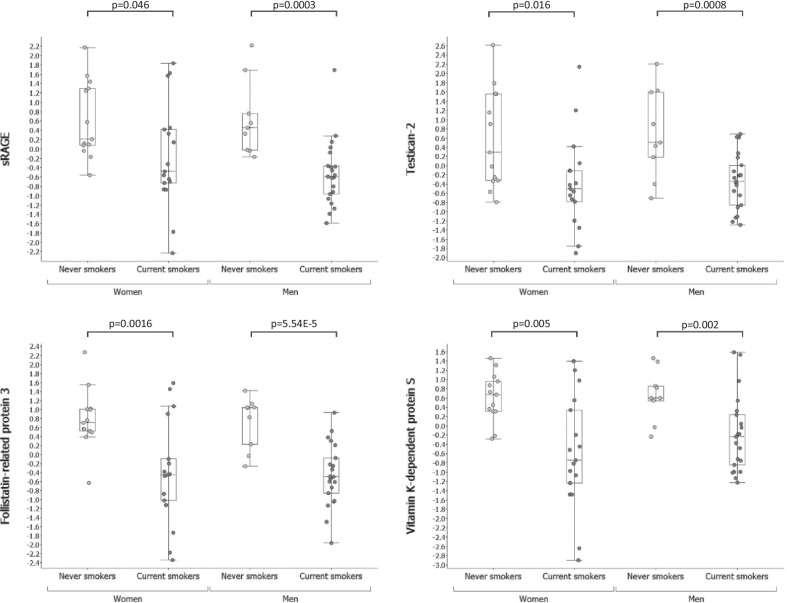
Abundance of sRAGE, testican-2, follistatin-related protein 3 and protein S in never and current smokers. Y-axis shows normalized abundance of protein levels (log_2_ transformation and normalization to mean 0 and variance 1). Box ranges from the 25th to the 75th percentile and median value is marked with dotted line. p-values from pairwise comparisons are shown over each box plot. Protein abundance data was adjusted for age and the investigator

**Fig. 3:**
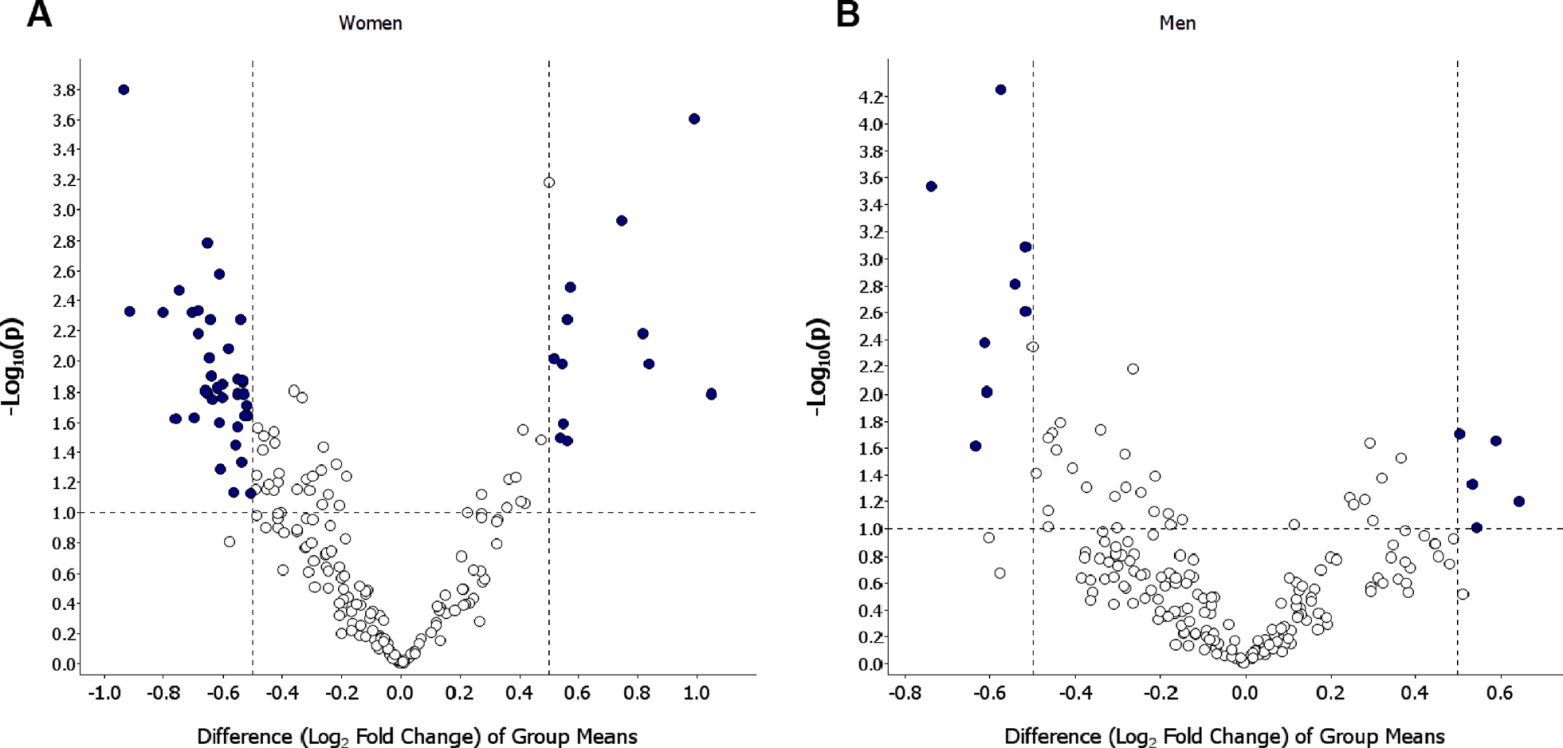
Differential abundance of proteins in female (**A**) and male (**B**) current smokers as compared to never smokers. The most prominent proteins for separating current smokers and never smokers are shown in the top left and top right of the plot and are coloured blue. The negative log10 of the p-value is plotted on the y-axis and the difference (log fold change) on the x-axis, based on the t-test between current and never smokers adjusted for age and the investigator

Active smoking exerted a significant impact on the protein composition of respiratory tract lining fluid (RTLF), with relative abundance of 81 proteins altered in CS compared to NS (p < 0.05, q < 0.124) (Table S2, Additional file 2). The majority of the differentially abundant proteins (62 proteins) were less abundant in CS compared to NS as exemplified by soluble receptor for advanced glycation end products (sRAGE), follistatin-related protein 3 (FSTL3), testican-2 (SPOCK2) and protein S (PROS1), which were among the proteins most clearly distinguishing CS from NS (Fig. 2). The proteins are described in more details in Table 2. This observation was true for both females and males (Fig. 3 and Table 3). We also observed significant differences in the effect of smoking on the RTLF protein profiles between females and males. When stratifying for sex, the relative abundance of 58 proteins was significantly altered in female CS as compared to NS (p < 0.05, q ≤ 0.167), while 27 proteins were significantly altered in male CS (p < 0.05, q ≤ 0.370; 8 with q < 0.2).

**Table 2:** Literature overview on selected proteins differentially abundant in current smokers as compared to never smokers

Protein name Entrez symbol (Uniprot ID)	Abundance in current smokers	Relevant literature findings, summary and reference
Advanced glycosylation end product-specific receptor, soluble	**↓**	Decoy receptor for RAGE (a pattern recognition receptor of the immunoglobulin super-family) [45, 67]
		RAGE signalling is a key driver of inflammation, oxidative stress and alveolar tissues damage in many pulmonary diseases, including COPD and is inhibited by sRAGE [45, 67]
sRAGE		Circulating sRAGE is decreased in COPD [45]
(Q15109)		Decrease in serum sRAGE is reported to occur already within 1 h after smoking, reaches its maximum after 8 h and is not fully restored even after 48 h [45]
Testican-2	**↓**	Forming part of the extracellular matrix
SPOCK2		It has been suggested to contribute to lung barrier function providing protection against influenza virus infection by restricting entry of the virus into epithelial cells [68]
(Q92563)		
		It is known to modulate matrix metalloproteinases expression and activation [48]
Follistatin-related protein 3	**↓**	A secreted glycoprotein structurally and functionally related to follistatin [69]
FSTL3		It binds and antagonises actions of members of the transforming growth factor beta (TGFβ) superfamily such as activin A (an important regulator of cigarette smoke-induced inflammation) [47, 69]
(O95633)		Follistatin, an activin A inhibitor, has been seen to be decreased in cigarette smoke-exposed human bronchial epithelial cells and administration of follistatin was found to attenuate cigarette smoke-induced airway inflammation in mice [47]
Protein S	**↓**	Involved in the inhibition of coagulation, clearance of apoptotic cells and inhibition of inflammation [70]
PROS1		In plasma, 30–40% of protein S exists in its free form and the remainder is forming a complex with C4b-binding protein (C4BP), an important regulator of complement activation by the classical pathway [70]
(P07225)		Protein S localizes C4BP to apoptotic cells where C4BP can down-regulate complement activation and therefore inhibit inflammation at the surface of apoptotic cells [70]
		Binding of free protein S to the surface of apoptotic cells also enhances phagocytosis of apoptotic cells by macrophages [71]
Adiponectin	**↓**	Anti-inflammatory adipokine that inhibits proinflammatory cytokines and induces anti-inflammatory cytokines [72]
ADIPOQ		Studies have shown that subjects with emphysema have increased levels of adiponectin in BAL fluid while current smokers without COPD have reduced levels of adiponectin in BAL fluid [72]
(Q15848)		
Beta-2-microglobulin	**↓**	Component of the class I major histocompatibility complex (MHC I)
B2M		Identified as a proaging factor [73]
(P61769)		Studies have shown that serum β2M is significantly elevated in patients with COPD and the expression of β2M is significantly higher in lung tissue of emphysema [73]
Heat shock protein 90	**↑**	Functions as a molecular chaperone and contributes to the folding, maintenance of structural integrity and proper regulation of a subset of cytosolic proteins [74]
HSP90		Plays an important role in the UPR ("unfolded protein response") [58]
(P07900)		The expression of heat shock proteins is increased in the alveolar epithelial cells exposed to cigarette smoke extract [62]
(P08238)		
Translationally-controlled tumor protein (Fortilin)	**↑**	A pro-survival molecule [61]
TCTP		Plays an important role in the UPR where it protects cells from the apoptotic cell death [61]
(P13693)		
Ferritin	**↑**	An iron-storage protein [6]
(P02794)		Increased levels could indicate iron overload [6]
(P02792)		Increased in serum and BAL fluid in current smokers [6, 75]
Macrophage mannose receptor 1	**↓**	A pattern recognition receptor found on M2 macrophages which are considered to have anti-inflammatory, wound-healing properties and are involved in the removal of apoptotic cells [76]
MRC1, CD206		Reduced expression of MRC1 has been associated with a reduced removal of apoptotic cells in COPD [63]
(P22897)		Lack of MRC1 may also result in upregulation of pro-inflammatory cytokines during endotoxin induced lung inflammation in mice [77]
Interleukin-1 receptor-like 1	**↓**	A receptor for interleukin-33 (IL-33) [64, 65]
IL1RL1, ST2		Exists in several isoforms [64, 65]
(Q01638)		The membrane-bound ST2 binds IL-33, inducing pro-inflammatory immune responses and cytokine production and subsequently eliciting airway inflammation [64, 65]
		The soluble form of ST2 functions as a decoy receptor neutralizing IL-33 activity and is therefore considered to have an anti-inflammatory function [64, 65]
Adenylate kinase isoenzyme 1	**↓**	Plays an important role in cellular energy homeostasis and in adenine nucleotide metabolism
AK1		
(P00568)

**Table 3:** Comparison of the most prominent proteins for separating current smokers from never smokers

Protein	Current smokers vs never smokers					
	Women			Men		
	p-value	q-value	log_2_(fold change)	p-value	q-value	log_2_(fold change)
Fibrinogen	1.59E−04	0.025	− 0.94			
Follistatin-related protein 3	1.64E−03	0.067	− 0.64	5.54E−05	0.011	− 0.58
Beta-2-microglobulin	4.72E−03	0.072	− 0.92			
Complement factor H	4.77E−03	0.072	− 0.81			
Complement C2	3.39E−03	0.072	− 0.76			
Complement C3b, inactivated	4.75E−03	0.072	− 0.71			
Vitamin K-dependent protein S	4.62E−03	0.072	− 0.69	2.47E−03	0.100	− 0.51
Complement C3	5.31E−03	0.072	− 0.64			
Inhibin beta A chain	2.65E−03	0.072	− 0.62	1.53E−03	0.078	− 0.54
Complement component C9	5.31E−03	0.072	− 0.54			
C3a anaphylatoxin des Arginine	6.54E−03	0.079	− 0.69			
Antithrombin-III	8.35E−03	0.094	− 0.58			
Complement decay-accelerating factor	0.016	0.097	− 0.67	4.20E−03	0.132	− 0.62
Adiponectin	0.016	0.097	− 0.67			
Complement C5	9.61E−03	0.097	− 0.64			
Hepatocyte growth factor-like protein	0.013	0.097	− 0.64			
Contactin-1	0.018	0.097	− 0.64			
Alpha-1-antichymotrypsin complex	0.015	0.097	− 0.62			
Complement factor D	0.017	0.097	− 0.60	0.010	0.216	− 0.60
EGF-containing fibulin-like extracellular matrix protein 1	0.014	0.097	− 0.60			
Fetuin-B	0.013	0.097	− 0.56			
Protein FAM3B	0.016	0.097	− 0.56			
Extracellular matrix protein 1	0.014	0.097	− 0.54			
Complement component C6	0.013	0.097	− 0.54			
Testican-2	0.016	0.097	− 0.54	8.08E−04	0.055	− 0.51
Immunoglobulin A	0.020	0.105	− 0.51	0.024	0.286	− 0.64
Complement C4	0.024	0.116	− 0.76			
Macrophage mannose receptor 1	0.024	0.116	− 0.69			
Complement component C7	0.023	0.116	− 0.54			
Lumican	0.023	0.116	− 0.51			
Extracellular superoxide dismutase [Cu–Zn]	0.025	0.118	− 0.62			
Pigment epithelium-derived factor	0.027	0.120	− 0.56			
Plasma kallikrein	0.036	0.134	− 0.56			
Advanced glycosylation end product-specific receptor, soluble	0.046	0.164	− 0.54	2.90E−04	0.029	− 0.74
Complement C4b	0.051	0.177	− 0.60			
Follistatin-related protein 1	0.073	0.198	− 0.56			
Immunoglobulin G	0.075	0.198	− 0.51			
Heat shock protein HSP 90-beta	2.50E−04	0.025	0.99			
Heat shock protein HSP 90-alpha/beta	1.18E−03	0.060	0.75			
14–3-3 protein zeta/delta	5.30E−03	0.072	0.57			
Translationally-controlled tumor protein	3.23E−03	0.072	0.58	0.022	0.286	0.58
Tropomyosin alpha-4 chain	6.61E−03	0.079	0.82			
14–3-3 protein beta/alpha	9.75E−03	0.097	0.52			
6-phosphogluconate dehydrogenase, decarboxylating	0.010	0.097	0.55	0.063	0.397	0.64
Heat shock cognate 71 kDa protein	0.011	0.097	0.84			
Ferritin	0.016	0.097	1.05			
Alpha-enolase	0.026	0.118	0.55			
Fatty acid-binding protein, heart	0.032	0.130	0.54	0.098	0.463	0.55
Protein S100-A6	0.033	0.130	0.57			
Cofilin-1				0.020	0.286	0.51
C-X-C motif chemokine 16				0.047	0.370	0.54

Proteins shown were selected based on the volcano plots shown in Fig. 3a and b

### Former smokers versus never smokers

When comparing FS to NS, the levels of 31 proteins were altered, with all proteins being less abundant in FS (p < 0.05, q ≤ 0.31; 14 with q < 0.2). Stratifying for sex, 7 proteins were altered in female FS (p < 0.05, q ≤ 0.96) and 9 proteins were altered in male FS (p < 0.05, q ≤ 0.77) as compared to NS. Although p-values were significant in the analysis stratified by sex, none of the proteins in females and only one protein in males were found to be significantly altered after correction for multiple testing (q-value < 0.2).

### Effects of smoking cessation on the protein profile of small airways

To determine whether the changes in protein profile caused by active smoking persist after smoking cessation, proteins altered in CS were compared to those remaining altered in FS compared to NS (Table 4). Among the 62 proteins less abundant in CS as compared to NS, four (MRC1, CD55, ST2, AK1) remained significantly less abundant in FS, while the levels of the rest of these proteins were more similar to the levels observed in NS. Of the 19 proteins more abundant in CS as compared to NS, none remained more abundant in FS as compared to NS, twelve of these proteins were found to be significantly less abundant in FS as compared to NS (Table 4), and seven proteins were present at the same levels in FS as in NS.

**Table 4:** Proteins significantly altered in both current and former smokers as compared to never smokers

Protein	Current smokers vs never smokers			Former smokers vs never smokers		
	p-value	q-value	log_2_(fold change)	p-value	q-value	log_2_(fold change)
Complement decay-accelerating factor	7.52E−05	0.002	− 0.64	4.63E−02	0.310	− 0.34
Macrophage mannose receptor 1	2.24E−02	0.084	− 0.40	2.03E−02	0.221	− 0.40
Interleukin-1 receptor-like 1	6.67E−03	0.039	− 0.36	2.68E−03	0.136	− 0.36
Adenylate kinase isoenzyme 1	4.43E−02	0.115	− 0.27	3.37E−02	0.260	− 0.20
Small ubiquitin-related modifier 3	4.01E−02	0.109	0.24	4.48E−02	0.310	− 0.18
Macrophage-capping protein	2.79E−02	0.092	0.33	3.23E−02	0.260	− 0.22
Peptidyl-prolyl cis–trans isomerase A	4.93E−02	0.124	0.36	5.95E−03	0.147	− 0.45
C-X-C motif chemokine 16	3.41E−02	0.096	0.37	1.34E−02	0.194	− 0.54
Cofilin-1	1.51E−02	0.061	0.38	1.21E−02	0.190	− 0.29
Gelsolin	1.46E−02	0.061	0.39	1.02E−02	0.189	− 0.32
Fructose-bisphosphate aldolase A	2.19E−02	0.084	0.43	2.30E−02	0.225	− 0.30
Ras-related C3 botulinum toxin substrate 1	2.33E−03	0.020	0.44	1.95E−03	0.136	− 0.25
14–3-3 protein zeta/delta	8.97E−03	0.047	0.44	2.96E−02	0.253	− 0.29
14–3-3 protein beta/alpha	7.38E−03	0.041	0.46	1.83E−02	0.221	− 0.27
6-phosphogluconate dehydrogenase, decarboxylating	1.13E−02	0.051	0.51	6.49E−03	0.147	− 0.38
Heat shock protein HSP 90-alpha/beta	1.97E−03	0.019	0.58	4.73E−02	0.310	− 0.22

Data presented is based on the t-test adjusted for age and the investigator

To further study the effect of smoking cessation on the small airway protein profile, the relationship between relative protein abundance and time since smoking cessation was assessed using linear regression adjusted for age. The relative abundance of 20 proteins in males and 12 proteins in females correlated with time since smoking cessation (p < 0.05). When only looking at proteins that were decreased in CS compared to NS, the abundance of 9 proteins increased with time after smoking cessation in male FS as compared to 6 proteins in female FS (Table 5).

**Table 5:** Association between protein levels and number of years since cessation of smoking in former smokers

Protein	Women (N = 24)		Men (N = 22)	
	p-value	R-statistic	p-value	R-statistic
Hepatocyte growth factor receptor			0.002	0.64
Interleukin-6 receptor subunit beta			0.002	0.63
Plexin-B2			0.006	0.58
Follistatin-related protein 1			0.009	0.55
Follistatin-related protein 3			0.020	0.50
Inhibin beta A chain			0.030	0.47
Advanced glycosylation end product-specific receptor, soluble			0.035	0.46
Complement decay-accelerating factor			0.038	0.46
Complement C2			0.047	0.44
Antithrombin-III	0.001	0.63		
Thyroxine-binding globulin	0.010	0.53		
Heparin cofactor 2	0.012	0.52		
Complement C3b, inactivated	0.020	0.48		
Complement component C9	0.020	0.48		
Pigment epithelium-derived factor	0.026	0.46		

Only proteins less abundant in current smokers compared to never smokers that significantly increase with time since smoking cessation are shown

p-value and R-statistic are based on linear regression adjusted for age

### Complement cascade

**Fig. 4:**
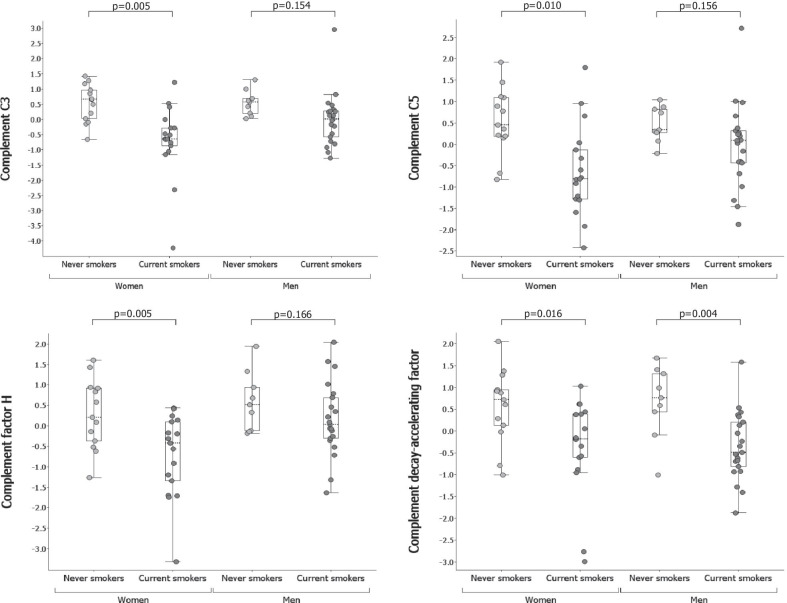
Abundance of C3, C5, complement factor H and complement-decay accelerating factor (CD55) in never and current smokers. Y-axis shows normalized abundance of protein levels (log_2_ transformation and normalization to mean 0 and variance 1). Box ranges from the 25th to the 75th percentile and median value is marked with dotted line. p-values from pairwise comparisons are shown over each box plot. Protein abundance data was adjusted for age and the investigator

Several complement factors were less abundant in female CS compared to NS (not observed in male). Of these, C3 and C5 were significantly less abundant in female CS (Fig. 4). No significant differences were observed in anaphylatoxin C5a between CS and NS in females (p = 0.891) or males (p = 0.672), but a higher C5a/C5 ratio was observed in female CS (log_2_(fold change) = 0.63, p = 0.006). Complement factors C2, C6, C7 and C9 were less abundant in female CS compared to NS, as well as factor D which was significantly less abundant in both female and male CS (Table 3). Factor H (Fig. 4) and factor I (log_2_(fold change) = − 0.43, p = 0.035) were also less abundant in female CS, and complement decay-accelerating factor (CD55) was less abundant in female and male CS compared to NS (Fig. 4 and Table 3).

## Discussion

**Fig. 5:**
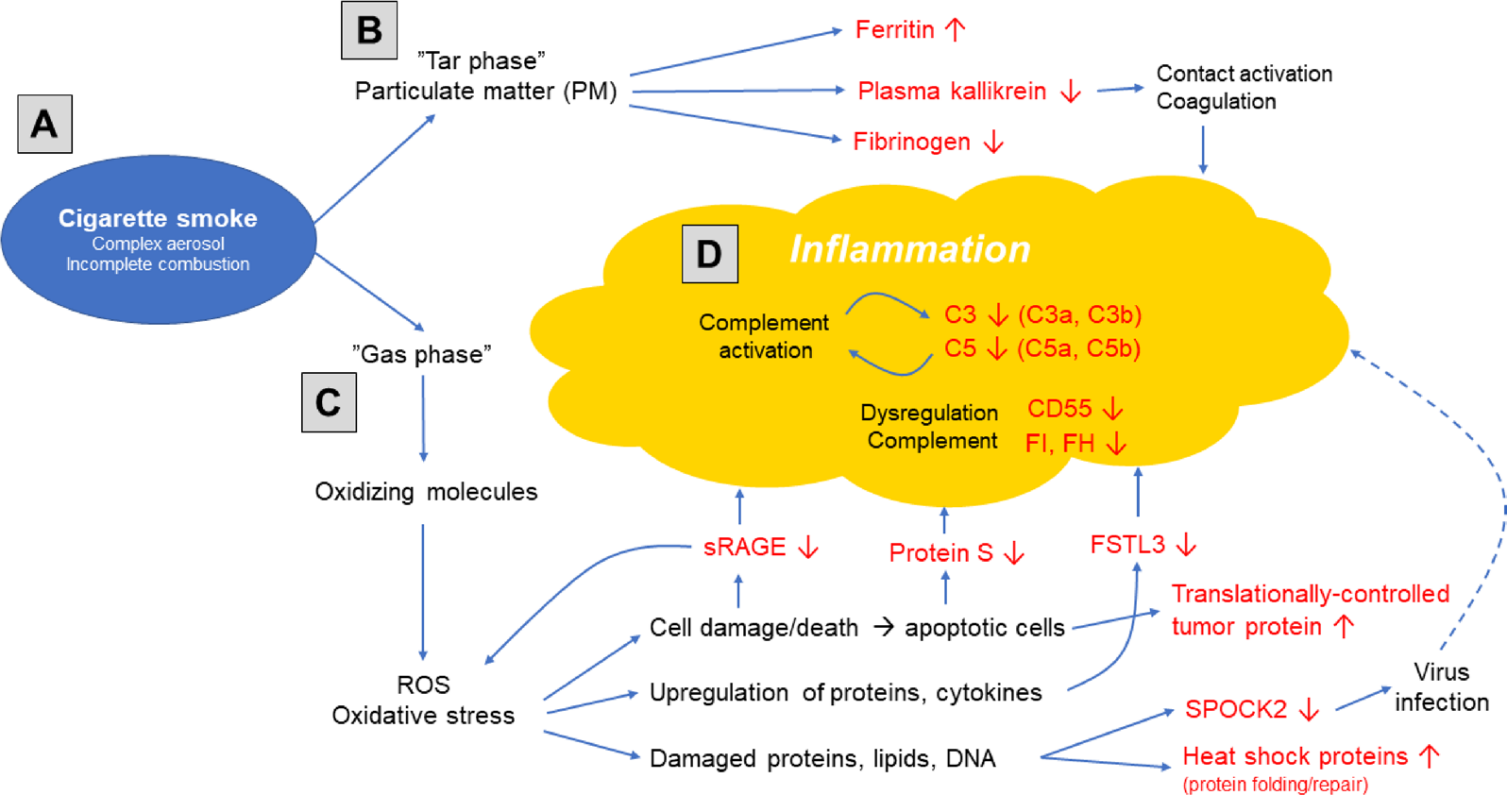
A model of the effects of cigarette smoke on the small airway protein profile. **A** Cigarette smoke consists of gaseous and particulate matter (tar) phase and the ROS in cigarette smoke induce oxidative stress. **B** Particulate matter causes an increase in ferritin levels in RTLF and affects fibrinogen and plasma kallikrein levels. Plasma kallikrein plays a role in the contact activation, coagulation and the alternative complement pathway. **C** Oxidative stress causes cell damage or apoptosis. Levels of TCTP, which protects cells from the apoptotic cell death, were increased in current smokers. Protein S, which is involved in the inhibition of coagulation and clearance of apoptotic cells was decreased in current smokers. In a complex with C4BP, protein S also prevents excessive complement activation and inflammation on the surface of apoptotic cells. sRAGE inhibits the induction of pro-inflammatory responses caused by the activation of RAGE signalling and decreased levels of sRAGE may contribute to inflammation. Upregulation of pro-inflammatory proteins and cytokines occurs due to oxidative stress and FSTL3 acts by neutralizing the activity of these proteins and induces the production of anti-inflammatory cytokines. Additionally, ROS cause damage to proteins, lipids and DNA and impair protein folding. Increased expression of heat shock proteins in the lungs exposed to cigarette smoke promotes the repair of misfolded proteins. Decreased levels of SPOCK2, which provides protection against influenza virus infection, may contribute to inflammation by making the epithelial cells more susceptible to viral infections. **D** Changes in the forementioned proteins all contribute to promoting inflammation and our findings suggest an important role of the complement system in this process. A decrease in CD55 and factors H and I, important inhibitors of complement activation, was observed in female current smokers, which could lead to excessive complement activation resulting in the depletion of C3 and C5, and this could be an important initiating step in the pathogenesis of small airway inflammation

In the present study we used a novel, non-invasive method to obtain biological samples from the small airways enabling assessment of the effects of smoking on the small airway proteome. Clear differences in the small airways protein profiles between never, former and current smokers were observed. These alterations were mainly detected in current smokers, while the protein profile of former smokers appeared to have returned to that observed in never smokers, with the exception of four proteins (MRC1, CD55, ST2, AK1) that remained downregulated. Further stratification revealed sex-associated differences with more pronounced alterations in female current smokers than in males. Based on observed changes in protein levels, the complement system was identified as being a biological pathway strongly affected by current smoking, particularly in female smokers, and a model of the small airway inflammation in smokers was extrapolated (Fig. 5).

The levels of 81 proteins were significantly altered in current smokers as compared to never smokers, and the proteins most clearly distinguishing current smokers from never smokers were soluble receptor for advanced glycation end products (sRAGE), testican-2 (SPOCK2), follistatin-related protein 3 (FSTL3) and protein S (PROS1). sRAGE has anti-inflammatory properties and deficiency of circulating sRAGE is associated with various human diseases. Decreased levels of plasma sRAGE have been previously observed in smokers with or without COPD, as compared to never smokers, and the levels were significantly correlated to lung function [44, 45]. In line with these observations, we observed significantly decreased levels of sRAGE in the lining fluid from small airways in current smokers, whereas, the levels of sRAGE in former smokers appeared to be similar to those in never smokers. These findings are consistent with decreased sRAGE levels being a marker of deficient inflammatory control, as previously suggested [46].

Levels of FSTL3, a protein structurally and functionally related to follistatin, were decreased in current smokers. Follistatin has anti-inflammatory properties and has been seen to be decreased in cigarette smoke-exposed human bronchial epithelial cells and administration of follistatin was found to attenuate cigarette smoke-induced airway inflammation in mice [47].

To our knowledge, differential abundance of testican-2 (SPOCK2) has not been previously reported to be associated with smoking or COPD. SPOCK2 is known to modulate matrix metalloproteinases expression and activation [48] with potential implications for small airway remodelling in context of COPD [49]. We did observe a slightly lower abundance of MMP-2 (also known as gelatinase A) in current smokers as compared to never smokers and a significant positive correlation between the levels of SPOCK2 and MMP-2 (not shown). Previous studies of the association between MMP-2 and smoking or COPD show conflicting results, some reporting increased MMP-2 expression in COPD patients [50–52], whereas others have observed decreased small airway levels of MMP-2 in COPD patients [53]. However, other metalloproteinases more often associated with COPD, such as MMP-9 and MMP-12, were not detected in this study.

Protein S is involved in the inhibition of coagulation, clearance of apoptotic cells and inhibition of inflammation. It binds and localizes C4BP, an important regulator of complement activation, to apoptotic cells where C4BP can down-regulate complement activation and therefore inhibit inflammation at the surface of apoptotic cells. We observed that protein S was significantly less abundant both in female and male current smokers as compared to never smokers. These findings suggest that the removal of apoptotic cells is compromised in the small airways of active smokers and are consistent with previous studies reporting the role of decreased clearance of apoptotic cells in the pathogenesis of COPD [54]. Furthermore, due to a decreased protein S in active smokers, the localization of C4BP to apoptotic cells may be diminished, which may result in excessive complement activation and inflammation in the small airways.

Our results show that active smoking in women causes the most pronounced alterations in the proteins related to the complement system. Female current smokers had decreased levels of the majority of the measured complement factors as well as decreased levels of complement factors H and I, key regulators of the alternative pathway (AP) preventing its excessive activation. Factor H also binds to apoptotic cells to limit the inflammatory potential of complement [55]. Furthermore, decreased levels of another important inhibitor of complement activation, CD55, were seen in the small airways of both female and male current smokers. Unlike the classical and the lectin pathways that are generally activated by recognition of exogenous material, the AP is constitutively active at a low level under normal conditions [56], and full activation is triggered by the presence of factor C3b and factor B which form a complex that eventually leads to the formation of AP C3 convertase that cleaves C3 into C3a and C3b, starting a positive feedback loop needed to commence AP activation. During inflammation, when cells are damaged, C3b is formed by the classical/lectin pathway, making the AP a powerful amplification mechanism of complement activation. Therefore, AP requires continuously active control mechanisms to maintain homeostasis [15]. Dysfunction of these regulatory proteins has been identified as a cause of several diseases [57], and decreased abundance of factors H and I in current smokers could potentially lead to chronic over-activation of the AP, which could be an important initiating step in the pathogenesis of small airway inflammation. As observed in current smokers, depletion of complement factors C3 and C5 as well as the concomitant increase in the pro-inflammatory mediator C5a (as C5a/C5 ratio), supports this hypothesis. The observed changes in the C5a/C5 ratio, but not in C5a levels, could be due to the nonspecificity of the SOMAscan aptamer for C5a, as it also binds to C5 albeit with approximately a three-fold lower affinity. A similar pattern was seen for C3a (as C3a/C3 ratio), however, SOMAscan aptamers for C3a are known to also bind to C3 with similar affinity and therefore the results could be influenced by the changes in C3. It has also been shown that exposure of C3 to cigarette smoke extract produces a functionally modified form of the molecule capable of activating the AP [16].

Heat shock protein 90 (HSP90) and translationally-controlled tumor protein (also known as fortilin or TCTP) were among the few proteins to be increased in current smokers as compared to never smokers. HSP90 plays an important role in the “unfolded protein response” (UPR) [58], a compensatory response elicited by impaired protein folding due to oxidative stress. The primary function of the UPR is to reduce the accumulation of aberrantly folded proteins and promote cell survival by increasing the expression of genes involved in protein chaperoning and folding, translation and degradation [59, 60]. However, when cells are damaged beyond repair, the UPR promotes elimination of afflicted cells through apoptosis [61]. Previous studies have also reported an increase in the expression of heat shock proteins in the alveolar epithelial cells exposed to cigarette smoke extract [62]. TCTP, a pro-survival molecule, has also been shown to play an important role in the UPR where it protects cells from the apoptotic cell death [61]. Our finding of increased HSP90 and TCTP levels in current smokers as compared to never smokers are consistent with previous studies suggesting cigarette smoke causes protein structural changes and impairs their folding which in turn results in the increased expression of the proteins involved in the UPR in order to promote cell survival.

The majority of alterations due to active smoking appeared to be reversible after smoking cessation. The former smokers had quit smoking at least 12 months prior to the start of the study, and time from smoking cessation ranged from one to 45 years, with a median of 11 years in females and 19 years in males. Only four proteins (MRC1, CD55, ST2 and AK1) remained less abundant in former smokers as compared to never smokers, indicative of permanent dysregulation. Reduced expression of MRC1 has been previously associated with reduced removal of apoptotic cells in patients with COPD, which may lead to lung tissue damage and COPD progression due to release of cytotoxic products from apoptotic cells [63]. ST2 is a receptor for IL-33. The membrane-bound ST2 binds IL-33, inducing pro-inflammatory immune responses, and the soluble form functions as a decoy receptor neutralizing IL-33 activity and is therefore considered to have an anti-inflammatory function [64, 65]. Persistent alterations of these proteins in smokers after smoking cessation could help explain why former smokers remain at an elevated risk for COPD despite quitting smoking. The proteins induced in current smokers were all less abundant in former smokers, some to levels even lower than observed in never smokers. These findings are consistent with an earlier observation that most cigarette smoke-related changes in the lung proteome are reversible [9].

Finally, we observed a surprisingly small overlap of proteins altered due to smoking in females and males, suggesting smoking affects women and men differently and by distinct mechanisms. It has previously been proposed that smoking may have more deleterious effects on lung health in women, and that female smokers may be more susceptible to developing COPD [24, 27]. Sex-specific differences in the BAL cell proteome between healthy smokers and smokers with COPD with more pronounced alterations in females have been reported [20, 23]. When comparing never smokers and smokers with normal lung function, one study reported a significant impact of smoking on the BAL cell proteome with alterations of more than 500 proteins (representing 15 molecular pathways) due to smoking. However, the majority of these alterations were sex-independent [66].

This study highlights alterations in the composition of the respiratory tract lining fluid caused by smoking, however, there are several limitations that need to be considered when interpreting the results. First of all, the sample size is small and an independent validation cohort is needed to confirm our findings. We also observed variability depending on the investigator performing the PExA sampling, and as a result the statistical analysis had to be adjusted accordingly. Never, former and current smokers were, however, examined in a random order and a systematic error therefore seems unlikely. Another potential cofounder was that never smokers were on average younger than the former smokers and current smokers, and therefore we adjusted the analysis for age. Choosing to include proteins in the analysis with RFU values > LOD in more than 50% of samples could be considered another possible limitation. However, due to the exploratory nature of the study, we chose to select 50% as the limit instead of the more commonly used 70–80%, to open the analytical window and explore proteins that could potentially be missed otherwise. For that same reason and also due to the small sample size, a more inclusive approach was taken and the changes were identified as significant at p-value < 0.05.

The observation that the majority of proteins with altered levels were less abundant in current smokers may be due to decreased protein expression in response to smoking. Another possibility that cannot be excluded is that smoke-induced changes in protein structure may compromise the affinity for the SOMAscan aptamers, leading to weaker signals. However, when comparing proteins detected in current smokers and never smokers, the overlap was significant, indicating that smoking status does not influence the SOMAscan analysis.

Another possible limitation is that the SOMAscan data is normalized based on the total amount of PEx and reported in relative values, which means that reported fold change values may not reflect the true magnitude of differences in the concentration of the differentially abundant proteins. Others ways to normalize the data were considered, such as normalization based on the total protein mass or to a housekeeping protein, but as PEx samples contain a small amount of biological material, it is difficult to measure the total protein concentration in order to use that for normalization. Additionally, the exhaled particle is a novel matrix and knowledge about potential housekeeping proteins that could be used for normalization is limited. Therefore, the data was instead normalized by diluting each sample prior to the SOMAscan analysis to equal concentrations of biological material as reflected by the total mass of the exhaled particles in each individual sample.

It will also be interesting to replicate and extend in a more severe COPD cohort to address whether the small airway proteome changes we observed in current smokers are present also in COPD patients and correlate with disease severity.

In conclusion, the study shows that smoking has a strong impact on protein expression in the small airways, and that smoking affects men and women differently. The observed protein alterations consistent with complement pathway activation in female smokers indicate that PExA proteomics can identify novel COPD disease processes as well as novel biomarkers. Thus, PExA sampling combined with high sensitivity protein analysis offers a promising platform for early detection of COPD and identification of novel COPD drug targets.

## Supplementary Information

**Additional file 1.** A detailed description of PEx sample preparation and analysis.

**Additional file 2: Table S1.** List of 203 proteins with RFU values > LOD in more than 50% of the samples that was used for further analysis. **Table S2.** Proteins significantly altered in current smokers as compared to never smokers in the joint model.

## Data Availability

The dataset used and analysed during the current study are available from the corresponding author on reasonable request.

## Abbreviations

COPD: Chronic obstructive pulmonary disease
RONS: Reactive oxygen/nitrogen species
PM: Particulate matter
RTLF: Respiratory tract lining fluid
CS: Current smokers
FS: Former smokers
NS: Never smokers
FEV1: Forced expiratory volume in 1 s
FVC: Forced vital capacity
MMEF: Maximal mid-expiratory flow
hsCRP: High-sensitivity C-reactive protein
PEx: Exhaled particles
RFU: Relative fluorescent units
LOD: Limit of detection
GO: Gene ontology
BAL: Bronchoalveolar lavage
AP: Alternative pathway
UPR: Unfolded protein response
