# Supplementary material for: Smoking induces sex-specific changes in the small airway proteome
Source: Respir Res. 2021 Aug 24;22:234. doi: 10.1186/s12931-021-01825-6 (PMC8385797; doi:10.1186/s12931-021-01825-6)
Supplement: Supplementary file 1 — Additional file 1. A detailed description of PEx sample preparation and analysis. [file 12931_2021_1825_MOESM1_ESM.docx]

**Supplementary material**

Smoking induces sex-specific changes in the small airway proteome.

Kokelj S, Östling J, Georgi B, Fromell K, Ekdahl KN, Olsson HK, Olin AC

## PEx sample preparation

Protein were extracted from the PEx substrate by adding sample buffer (proprietary SomaLogic SB17 buffer with addition of Tween-20 to 0.1% final concentration) to the centrifugal filter insert (Millipore Ultrafree-MC LH Centrifugal Filter) containing the PEx substrate membrane. The volume of sample buffer was adjusted to reach a final concentration of PEx at 1 µg/ml. After addition of sample buffer, the sample tubes were briefly vortexed and placed in a thermal rotary shaker for 1 h at 30 °C and 400 rpm. After incubation on the thermal shaker, protein extracts were retrieved in the lower part of the centrifugal filter unit by centrifugation at 10,000 ×*g* for 10 min. Protein extracts were stored at -80 °C before sent to SomaLogic (Boulder, Co, USA) for analysis. Blank samples were made by extraction of the material on PEx substrates subjected to the same sampling and sample preparation procedure as all other samples, omitting the breathing maneuver.

*SOMAscan analysis*

SOMAscan is a highly multiplexed, aptamer-based assay optimized for protein biomarker discovery, which is made possible by the simultaneous measurement of a broad range of protein targets. This assay measures 1305 human protein analytes and has proved successful in the identification of biomarker signatures in a variety of biomedical applications [1]. It utilizes synthetic DNA SOMAmers (Slow Off-rate Modified Aptamers) that bind to proteins with high affinity and specificity in a similar fashion to antibodies [2]. SOMAmer reagents are constructed with chemically modified nucleotides. The SOMAscan assay measures native proteins in complex matrices by transforming each individual protein concentration into a corresponding SOMAmer reagent concentration, which is then quantified by standard DNA hybridization. The assay takes advantage of SOMAmer reagents’ dual nature as both protein affinity-binding reagents with defined three-dimensional structures, and unique nucleotide sequences recognizable by specific DNA hybridization probes. The assay is performed under Good Laboratory Practice (GLP)-like quality systems [3]. The readout in relative fluorescent units (RFU) is directly proportional to the amount of target protein in the initial sample, as informed by a standard curve generated for each protein-SOMAmer pair [4].

*Statistical analysis*

Statistical analysis was performed using Qlucore Omics Explorer (Qlucore Omics Explorer 3.6 software, Qlucore AB, Lund, Sweden). General linear model (GLM), with each variable normalized to mean 0 and variance of 1, was used to determine differences in protein abundance between NS, FS and CS and all the analyses were adjusted for the investigator performing the PExA measurements, as well as the age of the subjects. When adjusting for (eliminating) one or more factors, Qlucore Omics Explorer subtracts the part of the data that can be explained by the factor(s). This is done by fitting a linear model with the factors to be eliminated as predictors, and retaining only the residuals (i.e., subtracting the part explained by the predictors). Differential protein abundance is then tested in a GLM with the adjusted protein levels as dependent variable and the NS/FS/CS groupings as independent variable. This is equivalent to a standard t-test of the protein abundance in the two groups [5].

The log_2_(fold change) was calculated from the difference (δ) between the arithmetic average over the first group (A_x_) and the arithmetic average over the second group (A_y_), δ=A(log(x)) – A(log(y)).

1. Candia J, Cheung F, Kotliarov Y, Fantoni G, Sellers B, Griesman T, Huang J, Stuccio S, Zingone A, Ryan BM, et al: **Assessment of Variability in the SOMAscan Assay.** *Scientific Reports* 2017, **7:**14248.

2. Kraemer S, Vaught JD, Bock C, Gold L, Katilius E, Keeney TR, Kim N, Saccomano NA, Wilcox SK, Zichi D, Sanders GM: **From SOMAmer-Based Biomarker Discovery to Diagnostic and Clinical Applications: A SOMAmer-Based, Streamlined Multiplex Proteomic Assay.** *PLOS ONE* 2011, **6:**e26332.

3. Gold L, Ayers D, Bertino J, Bock C, Bock A, Brody EN, Carter J, Dalby AB, Eaton BE, Fitzwater T, et al: **Aptamer-Based Multiplexed Proteomic Technology for Biomarker Discovery.** *PLOS ONE* 2010, **5:**e15004.

4. SomaLogic_Inc.: **SOMAscan Proteomic Assay Technical White Paper.** <https://somalogic.com/>; 2016.

5. Wichura MJ: *The coordinate-free approach to linear models / Michael J. Wichura.* Cambridge [England] ;: Cambridge University Press; 2006.
